# 2-Hydroxy-3-methylanthraquinone inhibits homologous recombination repair in osteosarcoma through the MYC-CHK1-RAD51 axis

**DOI:** 10.1186/s10020-023-00611-y

**Published:** 2023-01-30

**Authors:** Doudou Jing, Xuanzuo Chen, Zhenhao Zhang, Fengxia Chen, Fuhua Huang, Zhicai Zhang, Wei Wu, Zengwu Shao, Feifei Pu

**Affiliations:** 1 Department of Orthopaedics, The Second Hospital of Shanxi Medical University, Taiyuan, 030001 China; 2 Department of Orthopedics, Union Hospital, Tongji Medical College, Huazhong University of Science and Technology, Wuhan, 430022 China; 3 Department of Radiation and Medical Oncology, Zhongnan Hospital, Wuhan University, Wuhan, 430071 China; 4 Department of Orthopedics, Wuhan Hospital of Traditional Chinese and Western Medicine, Huazhong University of Science and Technology, Wuhan, 430022 China; 5 Department of Orthopedics, Wuhan No.1 Hospital, Tongji Medical College, Huazhong University of Science and Technology, Wuhan, 430022 China

**Keywords:** 2-Hydroxy-3-methylanthraquinone, DNA damage response (DDR), MYC, CHK1, RAD51

## Abstract

**Background:**

Osteosarcoma is a malignant bone tumor that usually affects adolescents aged 15–19 y. The DNA damage response (DDR) is significantly enhanced in osteosarcoma, impairing the effect of systemic chemotherapy. Targeting the DDR process was considered a feasible strategy benefitting osteosarcoma patients. However, the clinical application of DDR inhibitors is not impressive because of their side effects. Chinese herbal medicines with high anti-tumor effects and low toxicity in the human body have gradually gained attention. 2-Hydroxy-3-methylanthraquinone (HMA), a Chinese medicine monomer found in the extract of Oldenlandia diffusa, exerts significant inhibitory effects on various tumors. However, its anti-osteosarcoma effects and defined molecular mechanisms have not been reported.

**Methods:**

After HMA treatment, the proliferation and metastasis capacity of osteosarcoma cells was detected by CCK-8, colony formation, transwell assays and Annexin V-fluorescein isothiocyanate/propidium iodide staining. RNA-sequence, plasmid infection, RNA interference, Western blotting and immunofluorescence assay were used to investigate the molecular mechanism and effects of HMA inhibiting osteosarcoma. Rescue assay and CHIP assay was used to further verified the relationship between MYC, CHK1 and RAD51.

**Results:**

HMA regulate MYC to inhibit osteosarcoma proliferation and DNA damage repair through PI3K/AKT signaling pathway. The results of RNA-seq, IHC, Western boltting etc. showed relationship between MYC, CHK1 and RAD51. Rescue assay and CHIP assay further verified HMA can impair homologous recombination repair through the MYC-CHK1-RAD51 pathway.

**Conclusion:**

HMA significantly inhibits osteosarcoma proliferation and homologous recombination repair through the MYC-CHK1-RAD51 pathway, which is mediated by the PI3K-AKT signaling pathway. This study investigated the exact mechanism of the anti-osteosarcoma effect of HMA and provided a potential feasible strategy for the clinical treatment of human osteosarcoma.

**Graphical Abstract:**

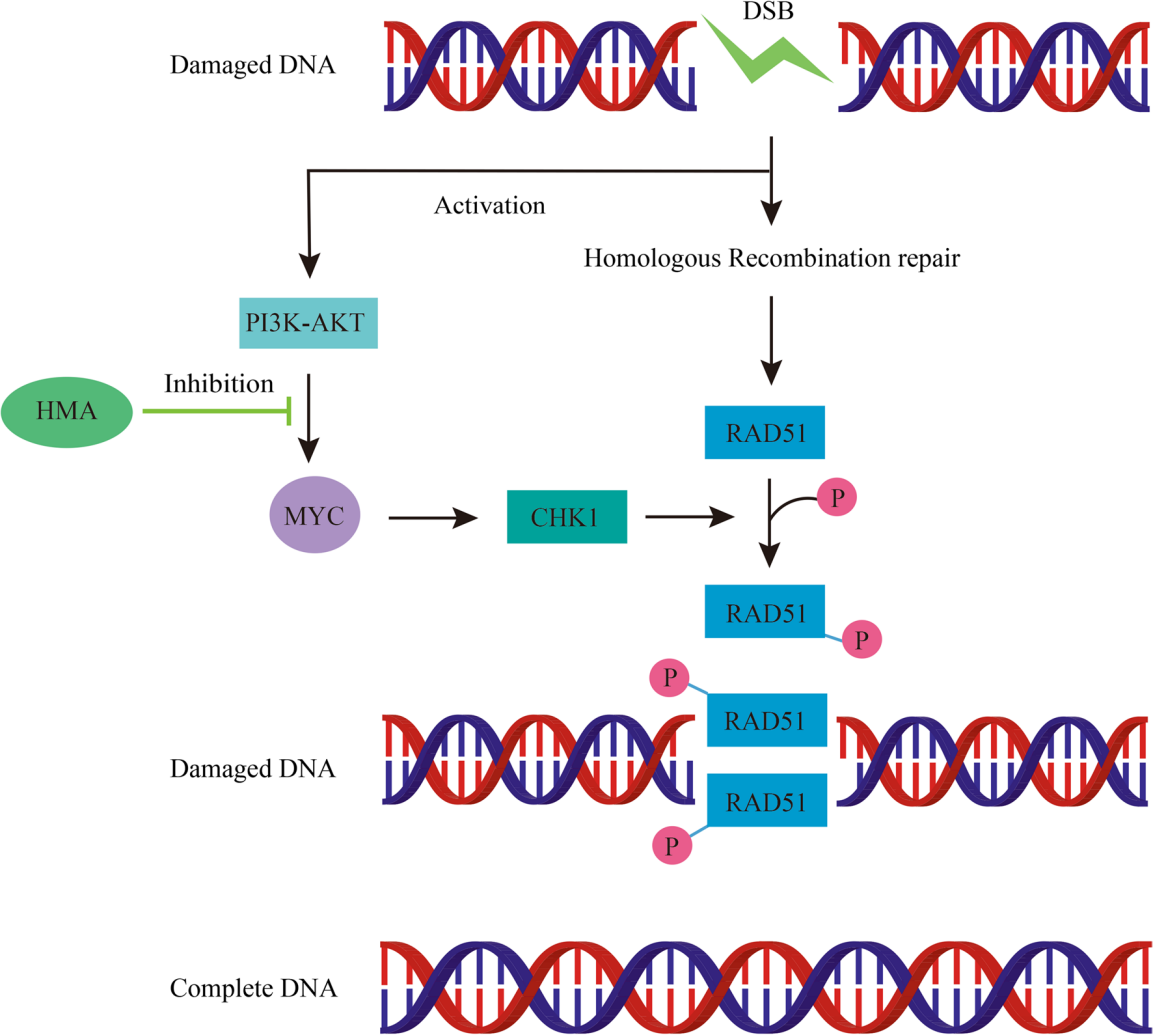

**Supplementary Information:**

The online version contains supplementary material available at 10.1186/s10020-023-00611-y.

## Introduction

Osteosarcoma (OS), one of the malignant bone tumors derived from mesenchymal cells with osteogenic potential and known for its rarity and genomic complexity, is the most common solid tumor affecting teenagers (Isakoff et al. 2015; Kansara et al. 2014). However, even with neoadjuvant chemotherapy and aggressive surgical resection, the 5-y survival rate for OS patients is approximately 60–70%. The low chemotherapy response rate is one of the important factors affecting the survival rate of these patients.

Research has shown that sensitivity to the cytotoxicity of systemic chemotherapy is primarily governed by the DNA damage response (DDR) pathway. In normal tissues, DDR maintains the stability of the genome and ensures normal progression of various life activities. However, in tumor cells, DDR protects them from toxic effects of radiotherapy and chemotherapy and promotes tumor initiation and progression (Maiorano et al. 2021; Cabrera-Andrade, et al. 2020). Targeting the DDR process is considered a feasible strategy benefitting cancer patients. OS with high levels of somatic structural variations and copy number alterations is characterized by chromosomal instability, in which chromosomes or parts of chromosomes are duplicated or deleted. The rarity and genomic complexity of OS have presented challenges in determining its molecular characterization and application of targeted therapies (Kansara et al. 2014). Therapies targeting the DDR are considered feasible strategies to solve these challenges of OS (Caracciolo et al. 2021; Caracciolo et al. 2020; Saitoh and Oda 2021). However, the clinical application of DDR inhibitors has not been impressive in OS patients.

Chinese herbal medicines with high anti-tumor effects and low toxicity in the human body have gradually gained attention. 2-Hydroxy-3-methylanthraquinone (HMA), a Chinese medicine monomer found in the extract of *Oldenlandia diffusa*, has significant inhibitory effects on various tumors (Gupta et al. 2004). However, its anti-OS effects and defined molecular mechanisms have not been reported. In this study, we found that HMA significantly inhibits OS proliferation and homologous recombination repair through the MYC-CHK1-RAD51 pathway, which is mediated by the PI3K-AKT signaling pathway. This study investigated the exact mechanism of the anti-OS effect of HMA and provided a potential feasible strategy for the clinical treatment of human OS.

## Materials and methods

### Cell lines and cell culture

MNNG/HOS and U-2 OS cells (purchased from Procell Life Science & Technology, Wuhan, China) were cultured in special cell line media (MEM containing 10% fetal bovine serum and 1% penicillin and streptomycin for MNNG/HOS, McCoy's 5A containing 10% fetal bovine serum and 1% penicillin and streptomycin for U-2 OS, also purchased from Procell Life Science & Technology).

### Acquisition of human tissue samples

This study was approved by the Ethics Committee of Union Hospital, Tongji Medical College of Huazhong University of Science and Technology, Wuhan, China, and conforms to the principles of the Declaration of Helsinki (2019-IEC-S274). All patients and their legal guardians provided written informed consent for human tissue acquisition in accordance with the National Regulations on the Use of Clinical Samples in China. The surgical specimen removed from the patient was sent to the Pathology Department of Union Hospital, Tongji Medical College of Huazhong University of Science and Technology. We subsequently obtained OS specimens from professional pathologists for further experiments.

### Cell viability

We detected OS cell viability after HMA treatment using the Cell Counting Kit-8 (CCK-8). Following the manufacturer’s protocol, 5 × 10^3^ cells were seeded in 96-well plates per well and incubated for 24 h before HMA treatment. A mixed solution containing 10 mL CCK-8 reagent (Beyotime, C0038, China) in 100 mL of media was added to the wells. After incubation for 2 h, the absorbance of the various samples was measured at 450 nm using a microplate reader.

### Colony formation assay

A colony formation assay was used to detect the proliferation capacity of OS cells after HMA treatment. Following this, 500 cells were evenly seeded in six-well plates and cultured for 2 weeks. Before staining with 0.1% crystal violet for 30 min, the samples were fixed with 4% paraformaldehyde for 10 min and washed three times with phosphate-buffered saline (PBS). Finally, images were captured using a camera. Colonies containing more than 50 cells were counted.

### Migration and invasion assay

Migration and invasion assays were conducted using a transwell chamber. First, 150 μL of medium containing 1% fetal bovine serum and 5 × 10^4^ cells was added to the upper compartments of the transwell chamber for the migration assay or a transwell chamber pre-coated with Matrigel for the invasion assay. Following this, 500 μL of medium containing 10% fetal bovine serum was introduced into the lower sections of the chamber and incubated for 24 h. These samples were then washed in PBS three times and fixed with 4% paraformaldehyde for 15 min. After washing three times with PBS, the samples were stained with 0.1% crystal violet for 30 min. After washing the samples with PBS and removing the cells in the upper chamber with a cotton swab, representative images were captured using an inverted microscope (Olympus, Japan).

### Annexin V-fluorescein isothiocyanate/propidium iodide staining

The death rate of OS cells after HMA treatment was detected with annexin V-fluorescein isothiocyanate (FITC)/propidium iodide (PI) staining. According to the manufacturer’s instructions, after collecting the cells, 200 μL of staining working solution containing annexin V- FITC and PI was added to re-suspend the cells. The mixture was incubated in the dark at 37 ℃ for 30 min before using a flow cytometer for detection. The proportion of PI-positive cells represents the rate of apoptosis.

### RNA sequencing

To further detect the mechanism of the inhibitory effect of HMA on OS, RNA sequencing was conducted. In total, 1 µg of RNA per sample was used as the starting material for RNA sequencing (RNA-seq). Sequencing libraries were generated using NEBNext Ultra RNA Library Prep Kit for Illumina (NEB, USA), according to the manufacturer’s instructions. Index codes were used to match the sequences to each sample. The samples were clustered on the cBot Cluster Generation System using the TruSeq PE Cluster Kit v3-cBot-HS (Illumina) according to the manufacturer’s instructions. Subsequently, libraries were sequenced on an Illumina NovaSeq platform, and 150-base pair (bp) paired-end reads were generated. The read numbers mapped to each gene were counted using Feature Counts, version 1.5.0-p3. Differential expressions (two biological replicates per condition) were analyzed using DESeq2 R package (version 1.16.1), and the statistical enrichment of differentially expressed genes in the Kyoto Encyclopedia of Genes and Genomes (KEGG) pathways was tested using the cluster Profiler R package.

### RNA interference

To further verify the mechanism of the inhibitory effect of HMA on OS, we used small interfering RNA (siRNA) to interfere with the expression of target genes. First, 10^6^ cells were seeded in six-well plates and cultured for 24 h. A mixture of Lipofectamine 3000 (Invitrogen, USA) and siRNA (Guangzhou Ruibo Biotechnology Co., Ltd.) was then added to the wells. After 16 h of infection, the medium was replaced with fresh medium. After verifying the interference efficiency, cells were collected for further experiments.

### Plasmid infection

To further verify the mechanism of the inhibitory effect of HMA on OS, we used plasmids to overexpress the target genes. First, 125 μL opti-MEM containing 3.75 μL Lipofectamine 3000 was added to 125 μL opti-MEM mixture containing 5 μg plasmid and 10 μL P3000™. After gentle mixing and incubating for 5 min at room temperature, the mixture was added to the cells at 60–70% cell confluence. After culturing for 72 h and verifying the infection efficiency, cells were collected for further experiments.

### Polymerase chain reaction

We used polymerase chain reaction (PCR) to detect the mRNA expression of target genes. Total RNA was extracted using TRIzol reagent (Invitrogen, USA) and transcribed into complementary DNA using a reverse transcription kit (Vazyme, China). Quantitative reverse transcription-PCR (qRT-PCR) was performed using the SYBR Premix Ex Taq II Kit according to the manufacturer's instructions (Vazyme, China). GAPDH was used to normalize the mRNA levels. The data were analyzed using the 2^−△△Ct^ method. The following primer sequences (from 5' to 3') were: MYC forward primer CGTCCTCGGATTCTCTGCTC and MYC reverse primer CTTCGCTTACCAGAGTCGCT.

### Western blotting

We used western blotting to detect the protein expression of target genes. Radio immunoprecipitation assay buffer mixture (Beyotime, P0013B, China) containing a protease inhibitor cocktail (Beyotime, P1006, China) was used to extract whole-cell lysates. The concentrations of these whole-cell lysates were determined using BCA protein assay kit (Beyotime, P0012, China). The whole-cell lysates from the samples were then separated via sodium dodecyl sulfate–polyacrylamide gel electrophoresis for 2 h. The proteins were transferred to 0.45-μm polyvinylidene difluoride (PVDF) membranes (Pierce Biotechnology, USA) at 400 mA for 20–40 min, wherein the timing depended on the size (kDa) of the target protein. After blocking with 5% skim milk powder in Tris-buffered saline with Tween™ (TBST) for 1 h at room temperature, the PVDF membranes were incubated with specific primary antibodies overnight at 4 °C. After washing with TBST three times, the membranes were incubated with horseradish peroxidase-conjugated secondary antibody (BOSTER, China) for 1 h. Finally, after treatment with enhanced chemiluminescence reagents (BOSTER, China), the images of the membranes were captured using the ChemiDoc XRS imaging system (Bio-Rad, USA).

### Immunofluorescence staining

To detect the subcellular structural localization and the protein expression of target genes, we conducted immunofluorescence staining assays. First, the samples were washed three times with PBS and then fixed with 4% paraformaldehyde for 15 min before being treated with 0.5% Triton X-100 (prepared with PBS) at room temperature for 20 min. The samples were then washed again three times with PBS. After then blocking for 30 min in 5% goat serum albumin, the samples were incubated overnight at 4 °C with H2A.X primary antibody (Abcam, USA). The cells were then washed with PBS-Tween™ three times and incubated with the corresponding secondary antibodies for 1 h in the dark. For counterstaining the samples in the dark, 4′,6-diamidino-2-phenylindole was used for 5 min. Samples were photographed using a fluorescence microscope (Olympus, Tokyo, Japan).

### Immunohistochemical staining

Before incubating with 3% H_2_O_2_ for 20 min and washing with water, paraffin sections were deparaffinized with different concentrations of xylene and alcohol. After using EDTA (pH 9.0) to recover the antigens and incubating with goat serum for 30 min, primary antibody was added to the sections and incubated overnight at 4 °C. The sections were then washed three times with PBS and incubated with a DAKO secondary antibody at room temperature for 30 min. The sections were later rinsed three times with PBS, incubated with DAB for 5–10 min, and washed again with PBS. Nuclei were then counterstained with hematoxylin for 5–10 min before sections were mounted and examined with an inverted microscope (Olympus, Japan).

### Chromatin immunoprecipitation–qPCR assay

To detect whether MYC could combine with the CHK1 promoter, we conducted a chromatin immunoprecipitation (CHIP)–qPCR assay. Cells were collected and centrifuged after fixation with a final concentration of 1% formaldehyde. The supernatant was removed from the samples, and the pellets were subjected to nuclear processing and chromatin shearing to yield 150–900 bp DNA fragments. The fragments were immunoprecipitated with the corresponding antibodies, and the chromosomes were eluted from the protein G microspheres and de-crosslinked. The DNA samples were then purified using spin columns, and the PCR assay was employed for CHIP enrichment efficiency analysis.

### Tumor xenografts

The procedures were approved by the Ethics Committee for Laboratory Animals of Huazhong University of Science and Technology (S2019009). In total, 10^7^ MNNG/HOS cells were collected and injected subcutaneously into the back of 8-week-old nude female mice (Laboratory Animal Centre, Huazhong University of Science and Technology). The tumor was measured with a caliper every 2 days. When the tumor volume reached 1000 mm^3^, the mice were euthanized. The animals were handled in strict compliance with the Guiding Principles for the Care and Use of Animals of the American Physiological Society. After tumors were collected, the growth curves of the tumors were plotted and the tumors were weighed.

### Statistical analysis

GraphPad Prism 9 software was used for statistical analysis, and the results are presented as means ± standard deviations. All data were tested for normality and homogeneity of variance using the Shapiro Wilk test, and the differing data were compared between the groups using the t-test or one-way analysis of variance. Statistical significance was set at a *p*-value < 0.05.

## Results

### HMA effectively inhibited the proliferation capacity of OS cells

**Fig. 1:**
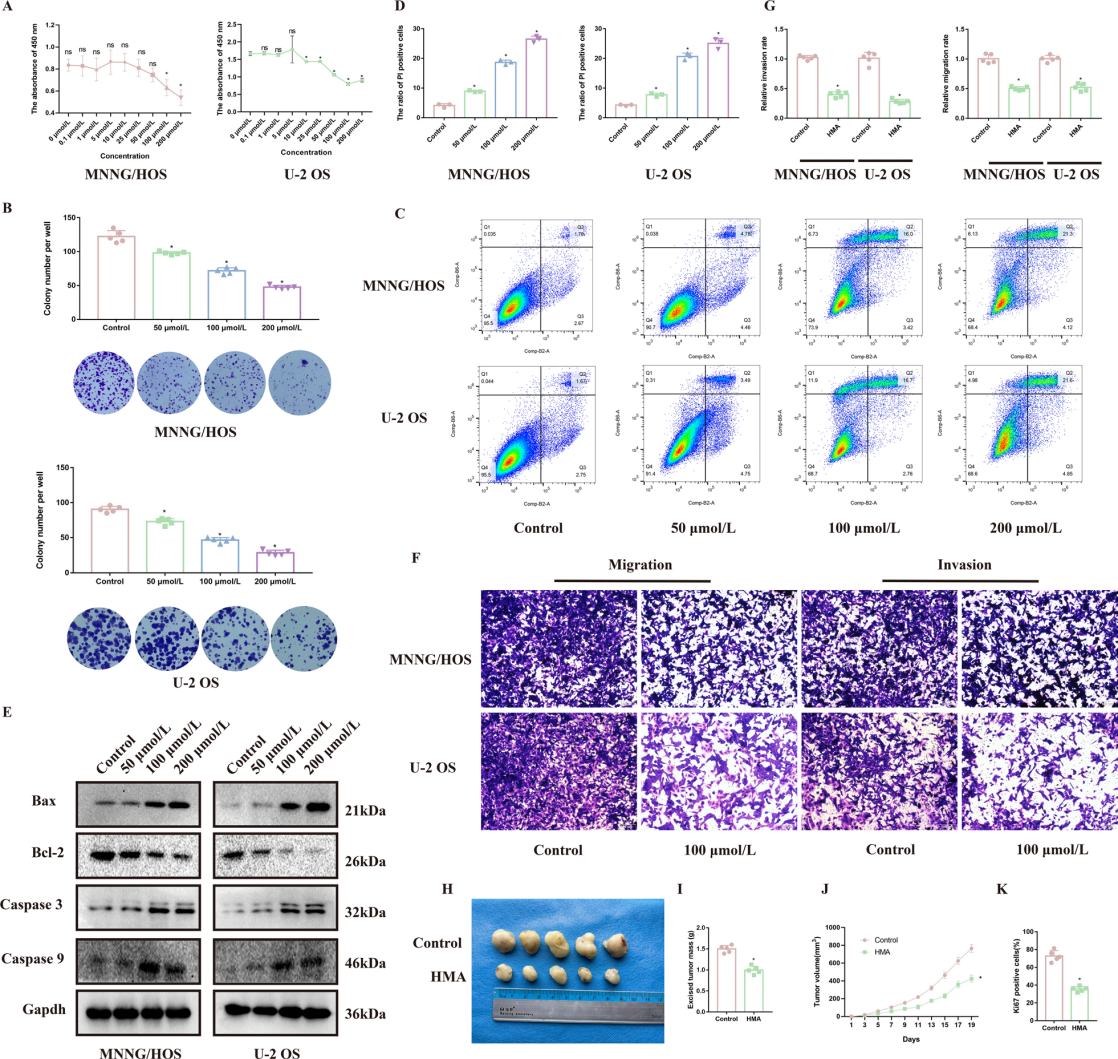
HMA effectively inhibits the proliferation capacity of osteosarcoma cells: The MNNG/HOS and U-2 OS cells were treated with different concentrations of HMA (0, 0.1, 1, 5, 10, 25, 50, 100, and 200 μmol/L) for **A** CCK-8 assay to evaluate the viability of the OS cells. Treatment with 0, 50, 100, and 200 μmol/L HMA of the OS cells was conducted for **B** colony formation assay, **C**, **D** Annexin V-FITC apoptosis detection assay, **E** Western blotting, and **F**, **G** transwell assay to detect the various biological behaviors of the OS cells in vitro. **H** The MNNG/HOS cells were injected subcutaneously into nude mice for xenograft assay. **I** Tumor growth curve and **J** excised tumor mass, as indicated. (**K**) IHC scores for Ki-67 staining, as indicated

The CCK-8 assay was performed to evaluate the anti-tumor effect of HMA on OS cells (Fig. 1A). The half-maximal inhibitory concentration (IC_50_) of HMA was then calculated (Additional file 1: Fig. S1A). HMA treatment inhibited the proliferative capacity of the MNNG/HOS and U-2OS cells in a dose-dependent manner. OS cells treated with HMA showed an obvious decrease in colony formation (Fig. 1B). HMA also increased the apoptosis rate of the OS cells, as shown by flow cytometry, and the protein related to the mitochondrial apoptosis pathway (Fig. 1C–E and Additional file 1: Fig. S1B). The invasion and migration capacities of the OS cells were also inhibited after HMA treatment (Fig. 1F–G). According to the above results, we chose 100 μmol/L instead of 200 μmol/L HMA for further experiments because of the more appropriate effect of promoting OS cell apoptosis with less toxicity and fewer side effects. In vivo, 100 μmol/L HMA treatment suppressed the mass and volume of the tumor and decreased the number of Ki-67-positive cells (Fig. 1H–K and Additional file 1: Fig. S1C). Thus, these results demonstrate that HMA inhibited the biological behavior of OS cells.

### MYC was highly expressed in OS patients

**Fig. 2:**
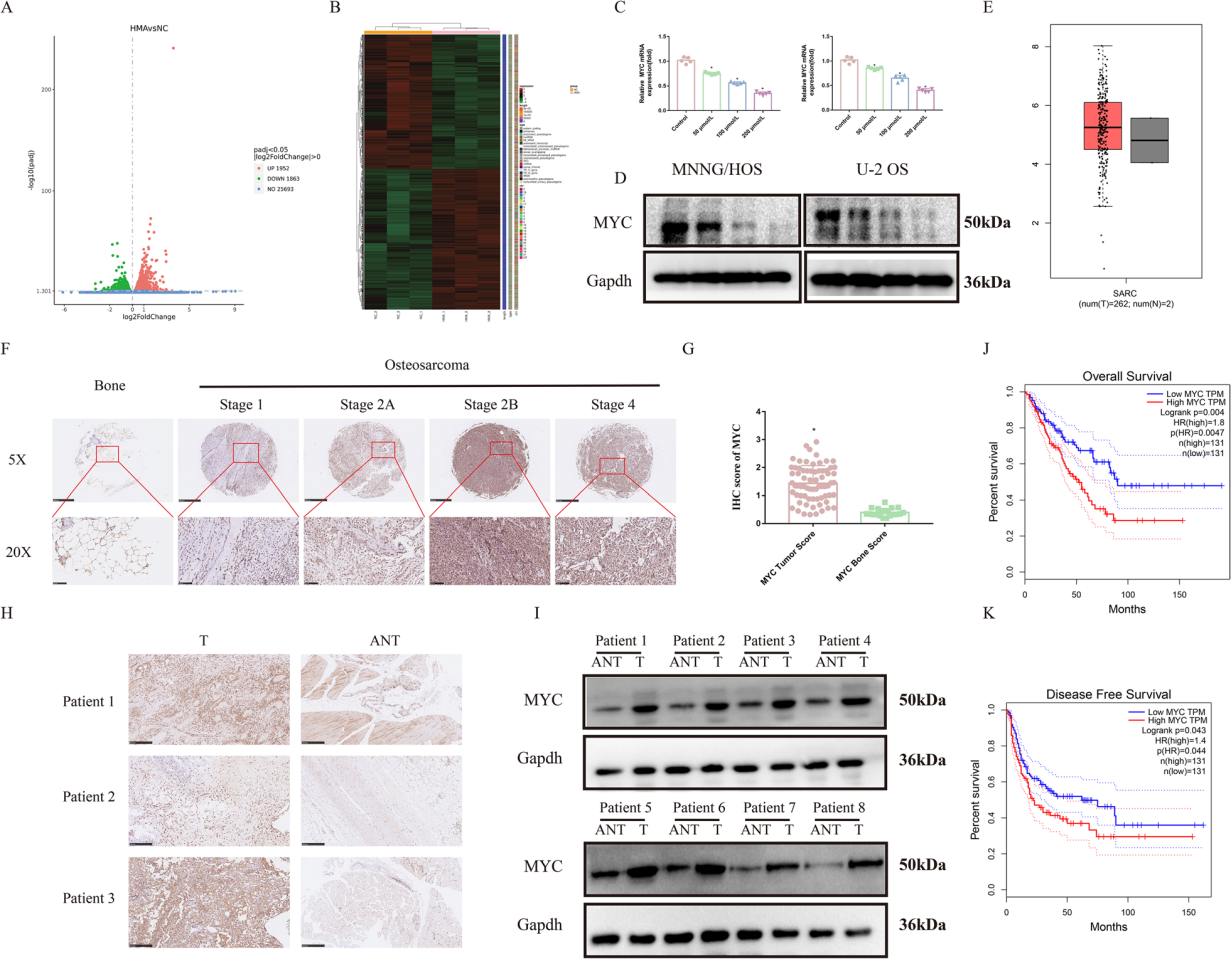
High expression of MYC in osteosarcoma patients: **A**, **B** The MNNG/HOS cells were treated with 100 μmol/L of HMA. After treatment, the cells were collected and subjected to RNA-sequencing analysis. Treatment with 0, 50, 100, and 200 μmol/L of HMA was conducted to detect MYC expression level in the OS cells by **C** PCR and **D** Western blot. **E** mRNA of MYC in OS patients. **F** MYC IHC staining of the OS and bone tissues microarray and **G** analysis of the IHC scores. **H**, **I** IHC staining and Western blotting for MYC of the tumor and tumor-adjacent normal tissue specimens. **J**, **K** Survival curve in OS patients

We employed RNA-seq analysis of HMA-treated MNNG/HOS cells to identify the underlying mechanism of HMA against OS. In total, 3815 differentially expressed genes were identified from three repetitive groups (Fig. 2A, B). The mRNA level of MYC was significantly downregulated after HMA treatment, according to the RNA-seq results. In fact, the protein and mRNA levels of MYC decreased gradually with increasing HMA concentrations (Fig. 2C, D and Additional file 1: Fig. S1D). MYC, a well-known oncogene, is involved in the occurrence, development, and prognosis of some tumors. To explore whether downregulation of MYC is related to the anti-OS effect of HMA, we first analyzed the role of MYC in OS patients. We observed that the mRNA expression level of MYC was significantly upregulated in OS patients compared to the normal controls, using the GEPIA web tool (http://gepia.cancer-pku.cn/) (Fig. 2E). The protein level of MYC was verified to be higher in the OS tissues than in the bone tissues via immunohistochemistry (IHC) staining in a tissue chip (OS specimens, n = 70; bone specimens, n = 10) (Fig. 2F, G). IHC staining and Western blotting of the tumor sections of our OS specimens also showed higher MYC protein levels than those in the tumor-adjacent normal tissue sections (Fig. 2H, I and Additional file 1: Fig. S1E). The overall survival curve and disease-free survival curve gain from GEPIA demonstrated that patients with high MYC expression had worse prognosis (Fig. 2J, K).

### Involvement of MYC in the anti-OS effect of HMA

**Fig. 3:**
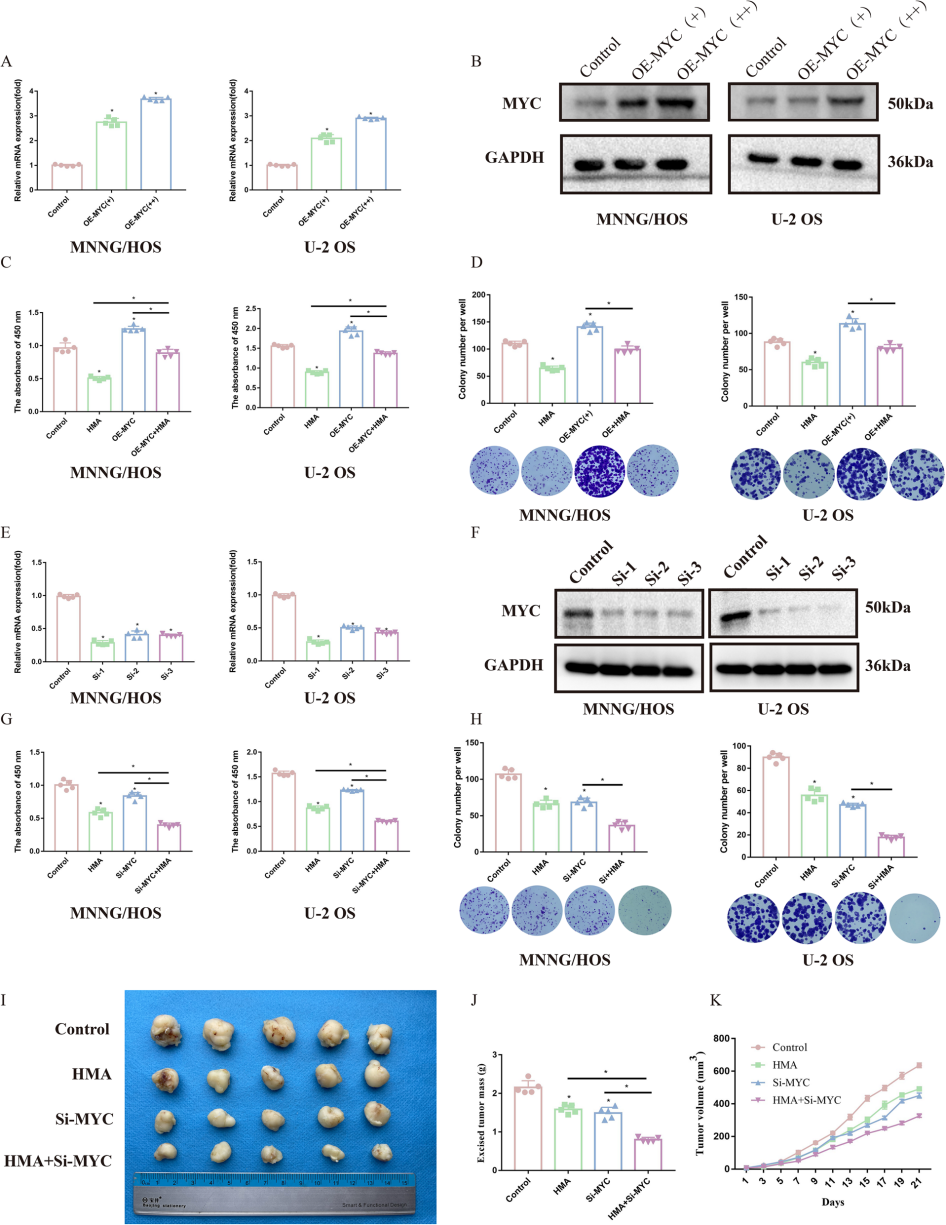
Involvement of MYC in the anti-OS effect of HMA: The OS cell lines were transfected with MYC overexpression plasmid and detected using **A** PCR and **B** Western blotting. The proliferation capacity of the OS cell lines after transfecting with MYC and HMA treatment was detected using **C** CCK-8 assay and **D** colony formation assay. The OS cell lines were transfected with si-MYC and detected using **E** PCR and **F** Western blotting. The proliferation capacity of the OS cell lines after transfecting with si-MYC and HMA treatment was detected using **G** CCK-8 assay and **H** colony formation assay. **I** MNNG/HOS cells were transfected with si-MYC and then injected subcutaneously into the nude mice for xenografts assay. **J** The tumor growth curve and (K) excised tumor mass as indicated

According to the results of RNA-seq, bioinformatics analysis, and clinical specimen verification, we found that MYC wa upregulated in OS tissues and unregulated after HMA treatment. Therefore, we further explored the role of MYC in the anti-OS effect of HMA. We found that, overexpression of MYC significantly impaired the anti-OS effect of HMA, as shown by the CCK-8 and colony formation assays (Fig. 3A–D and Additional file 1: Fig. S1F). In contrast, siRNAs targeting MYC showed a more significant anti-OS effect in these in vitro assays. The decrease of the IC_50_ of HMA also suggested that MYC may have mediated the HMA anti-OS effect (Fig. 3E–H and Additional file 1: Fig. S1G). In vivo, a xenograft assay demonstrated that MYC knockdown markedly enhanced the inhibitory effects of HMA on tumor growth, with decreased tumor mass, tumor volume, and ratio of the Ki-67-positive cells (Fig. 3I–K and Additional file 1: Fig. S1H, I). Thus, these results suggest that MYC induction plays an essential role in HMA-mediated anti-neoplastic effects.

### RAD51-mediated homologous recombination impaired after HMA treatment

**Fig. 4:**
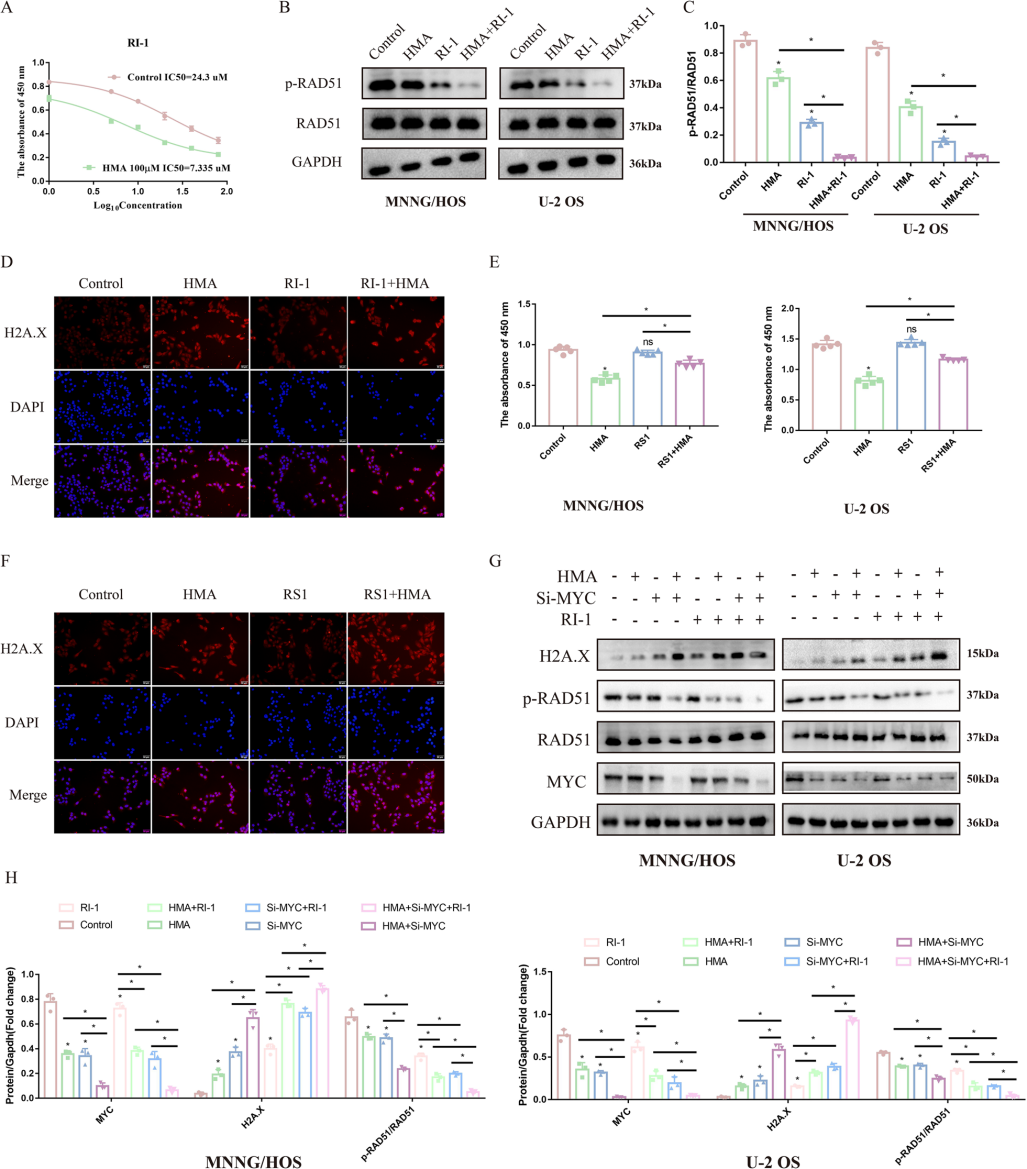
Impairment of the RAD51-mediated homologous recombination after HMA treatment: **A** The IC_50_ of RI-1 was detected in the MNNG/HOS cells with or without HMA treatment. **B**, **C** The expression of RAD51 and p-RAD51 was detected. **D** The subcellular structural localization of H2A.X was detected using immunofluorescence after RI-1 and HMA treatment. After treatment with RS-1 and HMA, the cells were analyzed using **E** CCK-8 assay and **F** H2A.X immunofluorescence. **G**, **H** Rescue assays show the relationship between HMA, MYC, and RAD51

We used olaparib (inhibitor of PARP1/2), NU7441 (inhibitor of the non-homologous end joining pathway), and RI-1 (inhibitor of homologous recombination repair) to explore which DDR pathway is involved in the impairment of HMA-induced OS proliferation. Compared with olaparib and NU7441, the RI-1 drug sensitivity (demonstrated by the IC_50_ values) of MNNG/HOS in the HMA treatment group decreased significantly, indicating that the RAD51-mediated homologous recombination repair process may participate in the anti-OS effect of HMA (Fig. 4A and Additional file 2: Fig. S2A, B). The active form of RAD51, phosphorylated at T309, decreased, whereas the nuclear localization of gamma-H2A.X (phospho S139), a marker of DNA double-strand breaks (DSBs), increased after HMA treatment (Fig. 4B–D). OS cells treated with HMA combined with RI-1, an inhibitor of RAD51, showed the lowest expression of RAD51 phosphorylated at T309 and the highest nuclear localization of gamma-H2A.X phosphorylated at S139 (Fig. 4D). RS1, the agonist of RAD51, increased cell viability and decreased nuclear localization, further confirming the role of RAD51 in the inhibition of OS by HMA (Fig. 4F). Additionally, rescue assays verified that the relationship between MYC and RAD51 mediated the homologous recombination repair (Fig. 4G, H). Thus, HMA-induced MYC downregulation led to a reduction in RAD51 phosphorylation, resulting in impaired OS proliferation.

### HMA impaired OS proliferation through the MYC-CHK1-RAD51 axis

**Fig. 5:**
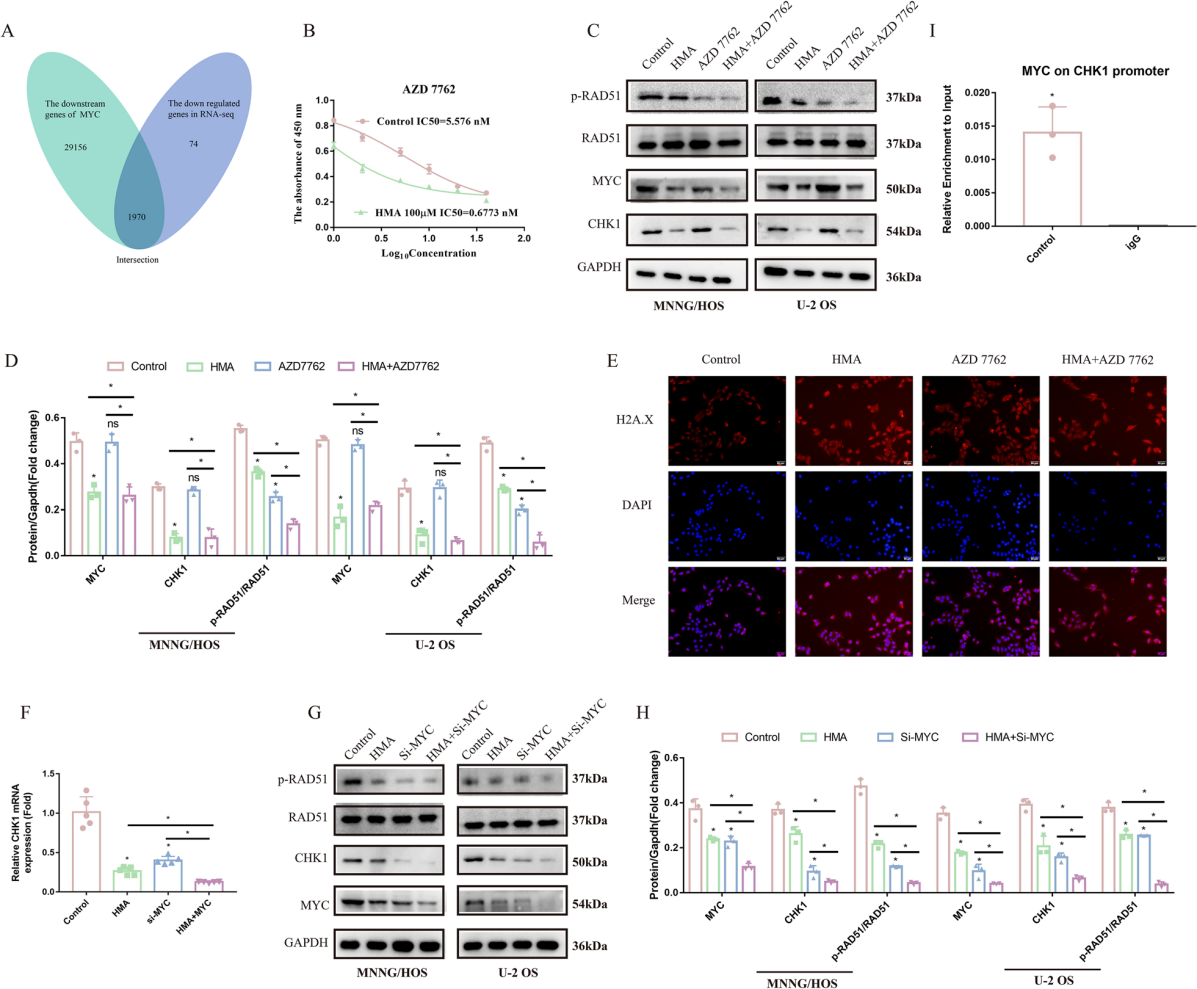
HMA impairs OS proliferation through the MYC-CHK1-RAD51 axis: **A** Venn diagram showing the intersection genes between the downstream genes of MYC and downregulated genes in RNA-sequencing. **B** The IC_50_ of AZD7762 was detected in the MNNG/HOS cells with or without HMA treatment. After treating with AZD7762 and HMA, the cells were analyzed using **C**, **D** Western blotting and **E** H2A.X immunofluorescence. After treating with si-MYC and HMA, the cells were analyzed using **F** PCR and **G**, **H** Western blotting. **I** CHIP–qRT-PCR assay was used to detect the enrichment efficiency of MYC on the CHK1 promoter

Although our results show that HMA-induced MYC downregulation leads to reduced phosphorylation of RAD51 and impaired OS proliferation, the exact underlying mechanism required further investigation. As a transcription factor, MYC does not phosphorylate the amino acid residues directly. Therefore, we predicted 30,946 genes regulated by MYC using the website tool GTRD (http://gtrd.biouml.org/#!). By combining these results with those of RNA-seq, we found that 1790 of the 30,946 predicted genes were downregulated after HMA treatment (Fig. 5A). Among them, the top 20 serine/threonine protein kinases with the largest content changes were selected to investigate which kinases could phosphorylate RAD51. MET, CHK1, and c-SRC were significantly downregulated, and notably, these were related to DDR (Luttich, et al. 2021; Bolland et al. 2021). Next, we detected the drug sensitivities (IC_50_ values) of these kinase inhibitors in the MNNG/HOS cells to identify the kinases that participate in the anti-OS effect of HMA. The results showed that the IC_50_ of AZD7762, a CHK1 inhibitor, decreased significantly (Fig. 5B and Additional file 2: Fig. S2C,D). Moreover, we found that AZD7762 further decreased the expression of RAD51 phosphorylated at T309 and increased the expression and nuclear localization of H2A.X (Fig. 5C–E). Additionally, in the group treated with HMA and si-MYC, the expression level of CHK1, regardless of the protein or mRNA levels, positively correlated with that of MYC (Fig. 5F–H). The results of the CHIP and PCR assays further verified that MYC initiated the transcription of CHK1 by binding to the CHK1 promoter (Fig. 5I). Thus, DDR was involved in the anti-OS effects of HMA through the MYC-CHK1-RAD51 signaling axis.

### Involvement of the PI3K-AKT pathway in the anti-OS effect of HMA

**Fig. 6:**
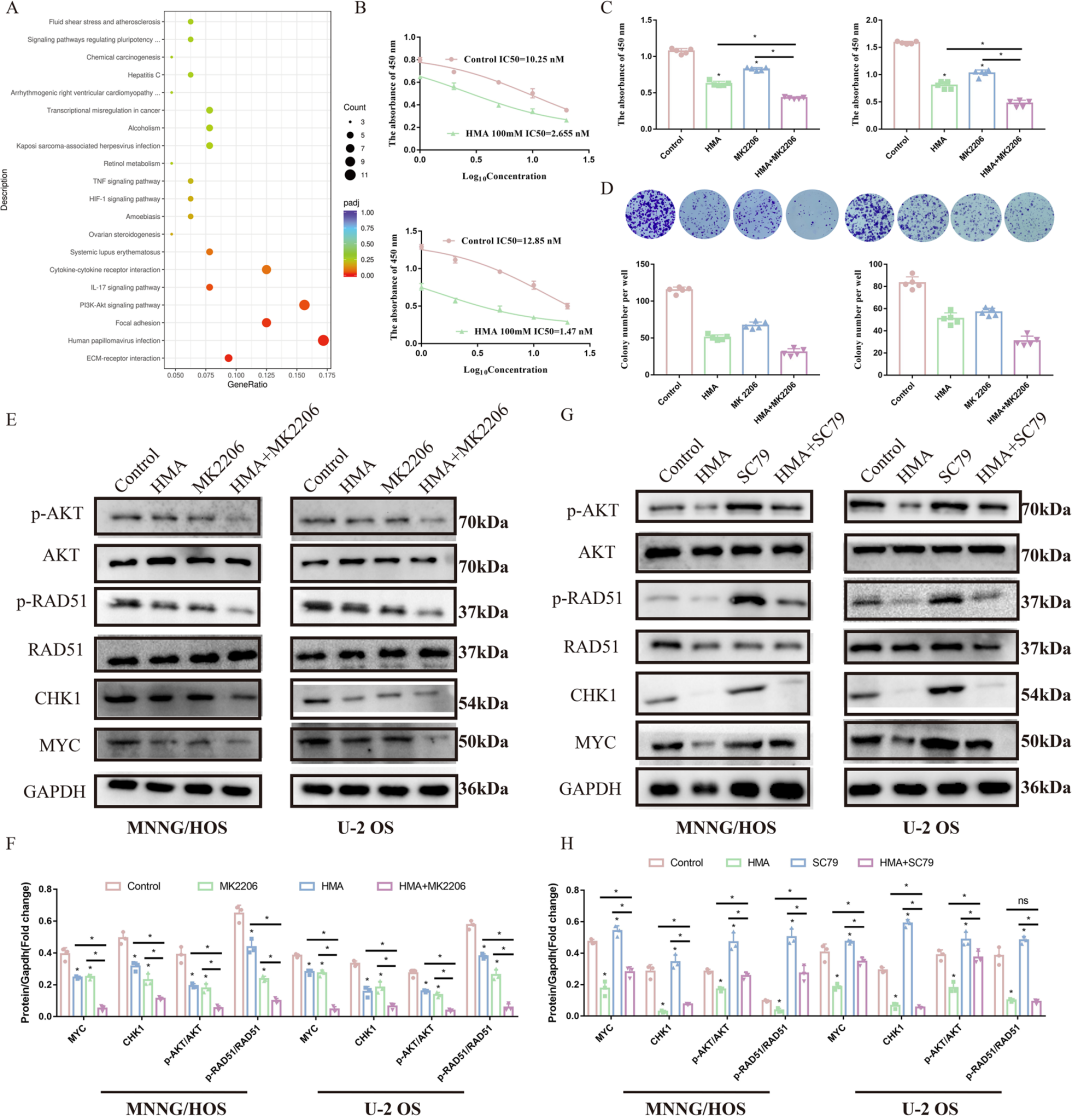
Involvement of the PI3K-AKT pathway in the anti-OS effect of HMA: **A** KEGG enrichment analysis based on the differential genes. **B** The IC_50_ of MK2206 was detected in the MNNG/HOS cells with or without HMA treatment. After treating with MK2206 and HMA, the cells were analyzed using **C** CCK-8 assay, **D** colony formation assay, and **E**, **F** Western blotting. **G**, **H** After treating with SC79 and HMA, the cells were analyzed using Western blotting

Enrichment analysis was conducted in the first 200 encoding protein genes that had the most obvious change in folding to identify the mechanism that downregulated the MYC-mRNA expression. KEGG enrichment analysis showed that the PI3K-AKT pathway was significantly altered after HMA treatment (Fig. 6A). Furthermore, we found that the IC_50_ value of MK2206, an AKT inhibitor, decreased significantly (Fig. 6B). Additionally, the proliferation capacity of the OS cells was severely inhibited by treatment with MK2206 and HMA compared with MK2206 or HMA alone (Fig. 6C, D). Thus, we can conclude that the PI3K-AKT pathway is involved in the anti-OS effect of HMA. Additionally, a rescue assay was used to determine whether HMA downregulated MYC expression through the PI3K/AKT signaling pathway. The results showed that in the group treated with MK2206, the expression of MYC and pRAD51 was significantly downregulated (Fig. 6E, F). In contrast, after SC79 treatment, an AKT agonist, the results showed upregulation of MYC and pRAD51 (Fig. 6G, H). Therefore, we conclude that HMA downregulates MYC through the PI3K-AKT-MYC pathway to inhibit the homologous recombination process.

## Discussion

Although the combination of neoadjuvant chemotherapy with aggressive surgical resection improved the 5-y survival of OS patients, high levels of structural variations in chromosomes and alterations in OS resulted in poor prognosis (Kansara et al. 2014; Mirabello et al. 2009). Abnormal DDR activity is one of the most common causes of structural variations in chromosomes and genomic heterogeneity. Research has reported that DDR-targeting strategies have unique advantages in anti-tumor therapy (Tang et al. 2021a; Andronikou and Rottenberg 2021). However, results from the application of DDR inhibitors in clinical trials have been less impressive because of the serious side effects reported in the body. The characteristics of low toxicity, high activity, and limited side effects make Chinese herbal medicines important in the field of anti-tumor therapy. HMA is one of the major ingredients of *Oldenlandia diffusa.* Reports have stated that HMA has an obvious anti-tumor effect, but its exact mechanism of action has not been reported. In this study, we found that HMA exerted a significant anti-OS effect. Specifically, HMA inhibited the homologous recombination repair in the OS cells through the MYC-CHK1-RAD51 axis, which is mediated by the PI3K-AKT signaling pathway (Graphical abstract).

MYC, located in the nucleus, is overexpressed in most human tumors, and it drives tumor development via different signaling pathways and at multiple levels. It belongs to the bHLH transcription factor family and combines with itself or other family members to form a dimer that acts as a transcription factor. Inhibitors aimed at obstructing the combination of dimers decrease tumor growth. MYC could also reactivate the transcription process that is mediated by other transcription factors by promoting the release of RNA polymerase II from a transcriptional pause at approximately 50 bp below the transcription initiation site. Notably, in tumors, MYC is found in the transcriptional pause site of more than 70% of genes that have a high transcription activity (Lustig et al. 2017; Rahl et al. 2010). In addition to promoting the expression of oncogenes, MYC inhibits the expression of some tumor suppressor genes, such as p15INK4B and p21Cip1, by combining with Miz-1 to promote tumor growth. Recently, high MYC expression has been reported to be associated with enhanced DDR in various tumors. MYC transcriptionally activates the genes related to DDR, such as PARP1/2, 53BP1, and BRCA1, which are associated with resistance to chemotherapy and radiotherapy (Fan et al. 2020). Additionally, tumors gaining higher MYC or MYC loci present more complex genome rearrangements and higher genomic instability (Chen, et al. 2018; Zhang et al. 2018). In this study, we found that MYC was highly expressed in OS patients. HMA inhibited OS proliferation by downregulating MYC expression through the PI3K-AKT signaling pathway.

The survival and reproduction of all organisms depends on the stable transmission of genetic material from generation to generation. In normal cells, inaccuracies in DNA replication drive genetic variations required for evolution. Excessive mutations lead to abnormal transcription and replication of cells, causing the cells to lose their stability and enter the process of senescence or death. However, in tumor cells, the genome is highly unstable, and the different mutations not only prevent the death of the tumor cells, but also lead to increased expression of oncogenes, loss of expression of tumor suppressor genes, and significant growth and metabolic advantages for the tumor cells. Genomic instability is a common feature of most tumors. Studies have shown that instability of the tumor genome endows tumor tissue with high heterogeneity and helps to effectively resist the efficacy of tumor therapy, including radiotherapy and chemotherapy. The genome of OS is highly unstable and characterized by duplicated or deleted chromosomes or parts of chromosomes (Ratnaparkhe, et al. 2019; Tang et al. 2021b). Research has reported that highly unstable genomic DNA in OS is associated with an abnormally enhanced DDR process. Genes related to DDR are also upregulated in OS (Ratnaparkhe, et al. 2019). The DDR includes blocking the cell cycle and initiating the DDR process. The proteins that play a role in cell cycle arrest differ according to the type of DNA damage. Various types of DDR exist, including base excision repair, nucleotide excision repair, DNA mismatch repair, and DNA DSB repair pathways. As shown in Fig. 4, we found that RI-1, an inhibitor of homologous recombination repair, significantly impaired the viability of the OS cells, suggesting that homologous recombination is involved in the HMA-induced anti-OS effect. Homologous recombination is a more precise DDR type of DNA DSBs, which mediates more accurate DNA repair using a template from a sister chromatid. Homologous recombination deficiency, such as that mediated by BRCA1 and BRCA2 (BRCA1/2) mutations, is associated with a high risk of breast cancer and ovarian cancer (Patel et al. 2021). RAD51, a key protein that mediates the homologous recombination repair pathway after DNA DSBs, is reportedly associated with low survival rates, high risk of tumor relapse, distant metastases, and chemotherapy resistance in breast cancer, lung cancer, brain cancer, and others (Laurini, et al. 2020; Lee, et al. 2019). In this study, HMA increased DNA damage by decreasing the activity of RAD51 (shown by the dephosphorylation of RAD51 at T309), inhibited OS proliferation, and promoted the rate of OS apoptosis.

CHK1, a serine/threonine protein kinase that mediates the pause in the cell cycle to initiate the DDR process, is highly expressed in various tumors and is associated with poor prognosis. When DNA damage occurs, CHK1 acts and integrates the signals from ATM and ATR and activates its downstream targets, such as CDC25A/B/C and P53, to block the cell cycle progression first and then activates some proteins related to DDR (Neizer-Ashun and Bhattacharya 2021). Currently, a considerable number of clinical trials have been conducted on several CHK1 inhibitors. According to previous reports, the use of the CHK1 inhibitor SCH900776 abolishes drug resistance in cells with high CHK1 abundance (David, et al. 2016). In multiple preclinical models of pediatric cancer, CHK1 inhibitor monotherapy was considered sufficient to overcome innate resistance or prevent acquired resistance. In OS, the therapy targeting CHK1, whether monotherapy or synergistic therapy, is regarded as a feasible strategy to overcome the difficulties encountered with chemotherapy (Heidler et al. 2020; Pandya, et al. 2020). In this study, after treatment with HMA, downregulated MYC decreased CHK1 expression at the transcriptional level, resulting in the inhibition of the RAD51-mediated homologous recombination process.

## Conclusion

HMA inhibited OS proliferation by impairing the DDR through the MYC-CHK1-RAD51 signaling axis, which is mediated by the PI3K-AKT signaling pathway. Thus, HMA treatment may be a feasible strategy benefitting OS patients.

## Supplementary Information

**Additional file 1: Fig. S1.** **A** The IC_50_ of HMA. **B** Statistical results of Fig. 1E. **C** Representative image captured after Ki-67 staining. **D** Statistical results of Fig. 2D. **E** Statistical results of Fig. 2I. (F) Statistical results of Fig. 3B. **G** Statistical results of Fig. 3F. **H** Representative image captured after Ki-67 staining and **I** the ratio of Ki-67-positive cells.

**Additional file 2: Fig. S2.** **A**–**D** The IC_50_ of inhibitors were detected in the MNNG/HOS cells with or without HMA treatment.

## Data Availability

The datasets generated during this study can be obtained by contacting the corresponding author with a reasonable request.

## References

[CR1] Andronikou C, Rottenberg S (2021). Studying PAR-dependent chromatin remodeling to tackle PARPi resistance. Trends Mol Med.

[CR2] Bolland H, Ma TS, Ramlee S, Ramadan K, Hammond EM (2021). Links between the unfolded protein response and the DNA damage response in hypoxia: a systematic review. Biochem Soc Trans.

[CR3] Cabrera-Andrade A (2020). Gene prioritization through consensus strategy, enrichment methodologies analysis, and networking for osteosarcoma pathogenesis. Int J Mol Sci.

[CR4] Caracciolo D (2020). Error-prone DNA repair pathways as determinants of immunotherapy activity: an emerging scenario for cancer treatment. Int J Cancer.

[CR5] Caracciolo D, Riillo C, Di Martino MT, Tagliaferri P, Tassone P (2021). Alternative non-homologous end-joining: error-prone DNA repair as cancer's achilles' heel. Cancers (basel).

[CR6] Chen LL (2018). SNIP1 recruits TET2 to regulate c-MYC target genes and cellular DNA damage response. Cell Rep.

[CR7] David L (2016). CHK1 as a therapeutic target to bypass chemoresistance in AML. Sci Signal.

[CR8] Fan L (2020). Histone demethylase JMJD1A promotes expression of DNA repair factors and radio-resistance of prostate cancer cells. Cell Death Dis.

[CR9] Gupta S, Zhang D, Yi J, Shao J (2004). Anticancer activities of *Oldenlandia diffusa*. J Herb Pharmacother.

[CR10] Heidler CL (2020). Prexasertib (LY2606368) reduces clonogenic survival by inducing apoptosis in primary patient-derived osteosarcoma cells and synergizes with cisplatin and talazoparib. Int J Cancer.

[CR11] Isakoff MS, Bielack SS, Meltzer P, Gorlick R (2015). Osteosarcoma: current treatment and a collaborative pathway to success. J Clin Oncol.

[CR12] Kansara M, Teng MW, Smyth MJ, Thomas DM (2014). Translational biology of osteosarcoma. Nat Rev Cancer.

[CR13] Laurini E (2020). Role of Rad51 and DNA repair in cancer: a molecular perspective. Pharmacol Therapeut.

[CR14] Lee JO (2019). Metformin overcomes resistance to cisplatin in triple-negative breast cancer (TNBC) cells by targeting RAD51. Breast Cancer Res.

[CR15] Lustig LC (2017). Inhibiting MYC binding to the E-box DNA motif by ME47 decreases tumour xenograft growth. Oncogene.

[CR16] Luttich L (2021). Tyrosine kinase c-MET as therapeutic target for radiosensitization of head and neck squamous cell carcinomas. Cancers (basel).

[CR17] Maiorano D, El Etri J, Franchet C, Hoffmann JS (2021). Translesion synthesis or repair by specialized DNA polymerases limits excessive genomic instability upon replication stress. Int J Mol Sci.

[CR18] Mirabello L, Troisi RJ, Savage SA (2009). Osteosarcoma incidence and survival rates from 1973 to 2004: data from the Surveillance, Epidemiology, and End Results Program. Cancer Am Cancer Soc.

[CR19] Neizer-Ashun F, Bhattacharya R (2021). Reality CHEK: understanding the biology and clinical potential of CHK1. Cancer Lett.

[CR20] Pandya PH (2020). Systems biology approach identifies prognostic signatures of poor overall survival and guides the prioritization of novel BET-CHK1 combination therapy for osteosarcoma. Cancers (basel).

[CR21] Patel PS, Algouneh A, Hakem R (2021). Exploiting synthetic lethality to target BRCA1/2-deficient tumors: where we stand. Oncogene.

[CR22] Rahl PB (2010). c-Myc regulates transcriptional pause release. Cell.

[CR23] Ratnaparkhe M (2019). Defective DNA damage repair leads to frequent catastrophic genomic events in murine and human tumors. Cancer Res.

[CR24] Saitoh T, Oda T (2021). DNA damage response in multiple myeloma: the role of the tumor microenvironment. Cancers (basel).

[CR25] Tang Z (2021). ATR inhibition induces CDK1-SPOP signaling and enhances anti-PD-L1 cytotoxicity in prostate cancer. Clin Cancer Res.

[CR26] Tang M, Bolderson E, O'Byrne KJ, Richard DJ (2021). Tumor hypoxia drives genomic instability. Front Cell Dev Biol.

[CR27] Zhang W (2018). Targeting the MYCN-PARP-DNA damage response pathway in neuroendocrine prostate cancer. Clin Cancer Res.

