# Supplementary figures and images for: 2-Hydroxy-3-methylanthraquinone inhibits homologous recombination repair in osteosarcoma through the MYC-CHK1-RAD51 axis
Source: Mol Med. 2023 Jan 30;29:15. doi: 10.1186/s10020-023-00611-y (PMC9887913; doi:10.1186/s10020-023-00611-y)

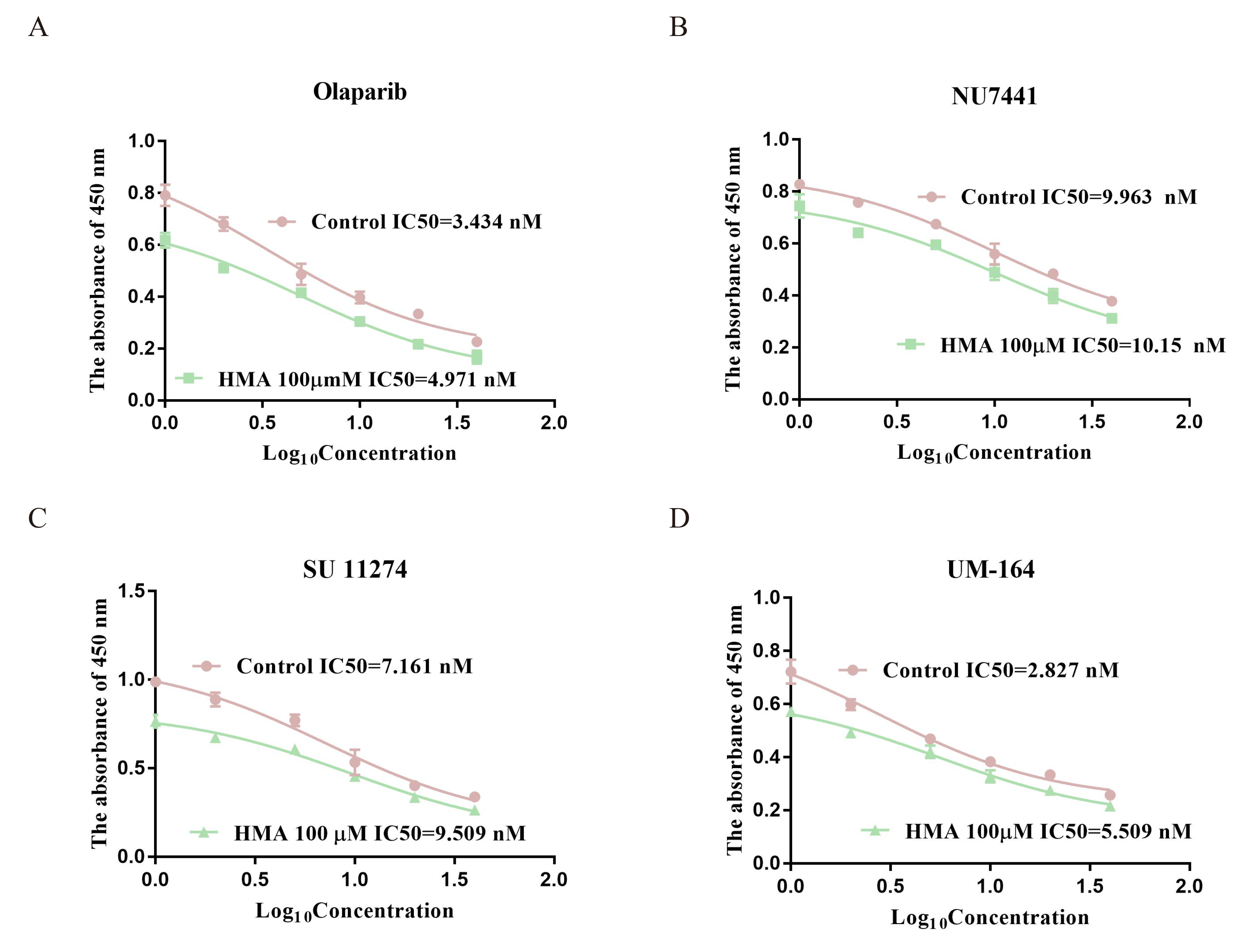

Supplement: Supplementary file 2 — Additional file 2: Fig. S2. A–D The IC50 of inhibitors were detected in the MNNG/HOS cells with or without HMA treatment. [file 10020_2023_611_MOESM2_ESM.tif]
